# KCl-induced cortical spreading depression waves more heterogeneously propagate than optogenetically-induced waves in lissencephalic brain: an analysis with optical flow tools

**DOI:** 10.1038/s41598-020-69669-6

**Published:** 2020-07-30

**Authors:** Buket Donmez-Demir, Şefik Evren Erdener, Hulya Karatas, Zeynep Kaya, Ilkay Ulusoy, Turgay Dalkara

**Affiliations:** 1 Institute of Neurological Sciences and Psychiatry, Hacettepe University, Ankara, Turkey; 2 Department of Electrical-Electronics Engineering, Faculty of Engineering, Middle East Technical University, Ankara, Turkey

**Keywords:** Optical imaging, Neurological models, Blood flow, Neurological disorders

## Abstract

Although cortical spreading depolarizations (CSD) were originally assumed to be homogeneously and concentrically propagating waves, evidence obtained first in gyrencephalic brains and later in lissencephalic brains suggested a rather non-uniform propagation, shaped heterogeneously by factors like cortical region differences, vascular anatomy, wave recurrences and refractory periods. Understanding this heterogeneity is important to better evaluate the experimental models on the mechanistics of CSD and to make appropriate clinical estimations on neurological disorders like migraine, stroke, and traumatic brain injury. This study demonstrates the application of optical flow analysis tools for systematic and objective evaluation of spatiotemporal CSD propagation patterns in anesthetized mice and compares the propagation profile in different CSD induction models. Our findings confirm the asymmetric angular CSD propagation in lissencephalic brains and suggest a strong dependency on induction-method, such that continuous potassium chloride application leads to significantly higher angular propagation variability compared to optogenetically-induced CSDs.

## Introduction

Cortical spreading depolarizations (CSD) are waves of intense neuroglial depolarization and subsequent suppression of neural activity that propagate slowly across the brain tissue accompanied by prominent hemodynamic changes^1^. They have been studied rigorously in rodents as well as swine, primates, and human beings because of their clinical significance in neurologic disorders like migraine and stroke. It was originally assumed that CSD waves spread homogenously and concentrically across the cortex^2, 3^. Later, irregular sulcal and gyral anatomical patterns in gyrencephalic brains have been shown to introduce heterogeneity to the spatiotemporal propagation of CSDs, causing radial, circling spiral or reverberating waves^4–6^. This brings up the question of whether the lissencephalic rodent brains may show a similar non-uniform propagation, considering their importance for experimental CSD research. It has already been shown that pathological peri-infarct depolarizations propagate non-concentrically, rather circling around the ischemic core in rodents that have agyrencephalic brains^7^. KCl-induced CSDs in mice and rats have also been shown to have heterogeneous CSD propagation patterns, modulated by cortical location, cortical depth, refractory periods, and number of wave repetitions^8–10^. Computational models and simulations have confirmed the refractory period-dependent modulation of this heterogeneous spatiotemporal propagation^11–13^.

Since CSDs are related to many neurological disorders like migraine, concussion, stroke, subarachnoid hemorrhage, disclosing the propagation features of these events is important to understand the clinical symptomatology that they cause. Appreciating the spatially heterogeneous consequences of repetitive CSDs is also important for experimental studies. Therefore, a relevant question is whether the spatiotemporal propagation variability is affected by the method of CSD induction. Today, novel optogenetic tools are available for CSD induction in addition to the other conventional methods like potassium chloride application and pinprick^14^. While CSDs are electrophysiological events, the hemodynamic changes strictly coupled to neuronal activity changes^15^ provide an opportunity to study their two-dimensional propagation profiles with appropriate optical imaging. Here in this study, we used laser speckle contrast imaging^16^ and automated optical flow analysis tools to systematically and quantitatively evaluate the variability in angular propagation of repeated CSDs^17^ in mouse brain, triggered by two different methods (topical potassium chloride application or optogenetic stimulation) with varying induction intervals. We present a comprehensive directional analysis of the spatiotemporal CSD propagation angles (i.e. statistical analysis of the propagation angles which are circular distributions with no true zero and with arbitrary high or low values), which are strongly dependent on the CSD triggering method.

## Methods

### Animals and surgical procedures

Animal housing, care, and application of experimental procedures were all done in accordance with institutional regulations as were approved by Hacettepe University Animal Ethics Committee (Approval numbers: 2011/18, 2013/66, and 2018/30). Experiments were conducted following the Guide for the Care and Use of Laboratory Animals and reported in compliance with the ARRIVE guidelines.

Male and female Swiss albino adult mice (25–35 g) (n = 7) (from Hacettepe University Experimental Animal Facility) and heterozygous Thy1-COP4/EYFP mice (n = 6) expressing light-activated channel Channelrhodopsin-2 fused to Yellow fluorescent protein in excitatory neurons (from Jackson Laboratories) were used. Animals were housed under diurnal lighting conditions (12 h darkness and 12 h light). Mice were anesthetized with isoflurane (1.5–2%) under continuous inhalation of oxygen (2 L/min) and were placed in a stereotaxic frame. Body temperature was monitored with a rectal probe and maintained at 37.0 ± 0.2 °C by a homeothermic blanket control unit. Pulse rate and oxygen saturation were monitored by an oximeter using a mini Y-clip hind paw probe.

The parietal bone was thinned using a microdrill (World Precision Instruments, USA) and a 1.5-mm burr hole was opened over the frontal region of the right hemisphere (1 mm anterior and 1 mm lateral to Bregma). The skull was irrigated with artificial cerebrospinal fluid (aCSF) at room temperature to reduce heating caused by the drilling procedure. Dura under the burr hole was kept intact and maintained moist by repeated applications of aCSF preheated to 37 °C until the experiment started.

CSDs were induced by topical potassium chloride (KCl) application over the dura in Swiss-Albino mice (n = 7). A cotton ball soaked with 1 M KCl was placed over the dura through the burr hole opened at the frontal region. The cotton ball was either left over the dura continuously or applied intermittently. In case of intermittent application (n = 3), the cotton ball was removed immediately upon detection of a CSD with laser speckle contrast imaging and another KCl-soaked cotton ball was applied exactly 10 min after the previous application. Each application resulted in a new CSD wave. We repeated this every-10 min-KCl application six times for each experiment. In case of continuous KCl application (n = 4), the cotton ball was left in place over the dura for 1 h and it was moistened with 1 M KCl with 5-min intervals to prevent drying.

For optogenetic stimulation experiments, six consecutive CSD waves were triggered with blue light in each animal at 5-min (n = 4 mice) or 10-min (n = 2 mice) inter-CSD intervals. 450 nm laser light was delivered with a fiberoptic probe for optogenetic stimulation, which was positioned and secured over the skull (1 mm anterior and 1 mm lateral to the bregma, the same location of the burrhole opening in KCl group) by a cable holder. No burr-hole drilling or thinning was performed in the skull for optogenetic stimulation to ensure minimal invasiveness. The optical fiber, 400 µm in diameter, with a numerical aperture of 0.48, was in full contact with the skull. Monte Carlo simulation for light propagation for this fiber placed directly over the skull was performed with previously published MATLAB functions^18^, using absorption coefficient 1.7 and scattering coefficient 2.8 as reported for mouse skull^19^. A suprathreshold light stimulus of 50 mJ was continuously applied for 10 s to trigger each CSD. Laser light was turned off upon completion of 10-s stimulus. This stimulation protocol has been optimized in our laboratory previously and reliably results in a CSD with every single application.

All CSDs, triggered by topical KCl or optogenetic stimulation, were confirmed by real-time laser speckle contrast imaging, with their typical blood flow changes and the blood flow data were recorded for offline analyses, as detailed below.

### Laser speckle contrast imaging

Laser speckle flowmetry was used to detect the cortical blood flow changes observed through the imaging window as described previously^16^. A 785 nm-laser diode (Thorlabs, USA) illuminated the brain surface through the thinned-skull window. A CCD camera (Basler acA1300-60gmNIR, Basler AG, Ahrensburg, Germany) attached to a stereomicroscope and custom-made software (kind gift of A.K. Dunn from The University of Texas at Austin) was used to obtain images. The field of view was adjusted as ~ 4 × 4 mm (960 × 960 pixels). Fifteen raw speckle images were acquired at 50 Hz (an image set), with 5 ms camera exposure time and averaged together. An image set was acquired every 10 s and a spatial speckle contrast (K) image was computed for each averaged image set, using a sliding grid of 7 × 7 pixels. Speckle contrast images were then converted to integrated correlation time (ICT) images (1/K^2^) to get blood flow indices, which were then processed further for optical flow analysis.

### Image processing and optical flow analyses

Image preprocessing and optical flow analyses were performed by using the MATLAB code by Afrashteh et al.^17^. The ICT image time series were first preprocessed for the optical flow analyses to be performed. First, the 960 × 960 pixel images were downsampled to 120 × 120 pixels with bilinear interpolation to optimize computer memory allocation. Then, the images were processed to determine the percentage change from baseline (ΔI/I_0_) for each pixel, to reduce regional bias caused by baseline blood flow heterogeneity. Individual sequences were then spatially filtered by a 5 × 5 pixel median filter and then by a 0.03 Hz temporal low-pass filter to reduce high-frequency noise in image series due to heartbeat or motion artifacts.

Next, using the combined local–global (CLG) optical-flow method, velocity vector fields were determined by estimating the displacement of pixels over time^17^. Magnitude and direction of the velocity vector for each pixel represented the speed and direction of motion. The reader is referred to the original paper^17^ for mathematical details of the CLG optical flow estimation approach. Briefly, CLG combines Horn–Schunk (HS) and Lucas–Kanade (LK) methods which are based on minimization of an energy function for the purpose of motion vector estimation and smoothness, respectively. “α” parameter is the weight of LK function over the HS function in the optimization problem. A hierarchical implementation is applied from coarse solutions to higher resolutions. Coarse solutions are obtained by downsampling the image sequences and parameter “ratio” specifies the downsampling ratio and parameter “minimum width” specifies the coarsest image size. Iteration parameters are for the number of iterations applied at different hierarchical levels. Although these parameters should be high for better convergence of the numerical solutions to the actual value, the cost of the computation time is a limiting factor. We took α = 0.08, ratio = 0.5, minimum width = 15, nOuterFPIterations = 50, nInnerFPIterations = 10, nSORIterations = 50. These parameters were chosen with a trial-and-error approach by using the algorithms in preliminary experiments and visually checking the code-generated vectors.

### Statistical methods

Average propagation vectoral angle for each laser speckle frame was calculated from the processed data and the data was loaded into CircStat MATLAB toolbox for statistical analysis of the directional data^20^. For each directional series, we computed the angular mean (and confidence interval), angular deviation, angular variance and, kurtosis, along with an Omnibus analysis on the uniformity of the angular distribution of framewise mean propagational vectors for each CSD. For intra-CSD comparisons (within the same CSD wave), directional data time series from each CSD wave was analyzed internally. For inter-CSD comparisons, average directional vectors of CSD propagation was analyzed among the 5 consecutive CSD waves except for the first CSD. Variance data were compared among the experimental groups with Kruskal–Wallis One-Way ANOVA test, followed by post-hoc Mann Whitney U test. The number of CSDs included for inter-CSD variance yield ~ 99% statistical power for α = 0.05 (two-tailed). p ≤ 0.05 was accepted as statistically significant.

## Results

**Figure 1:**
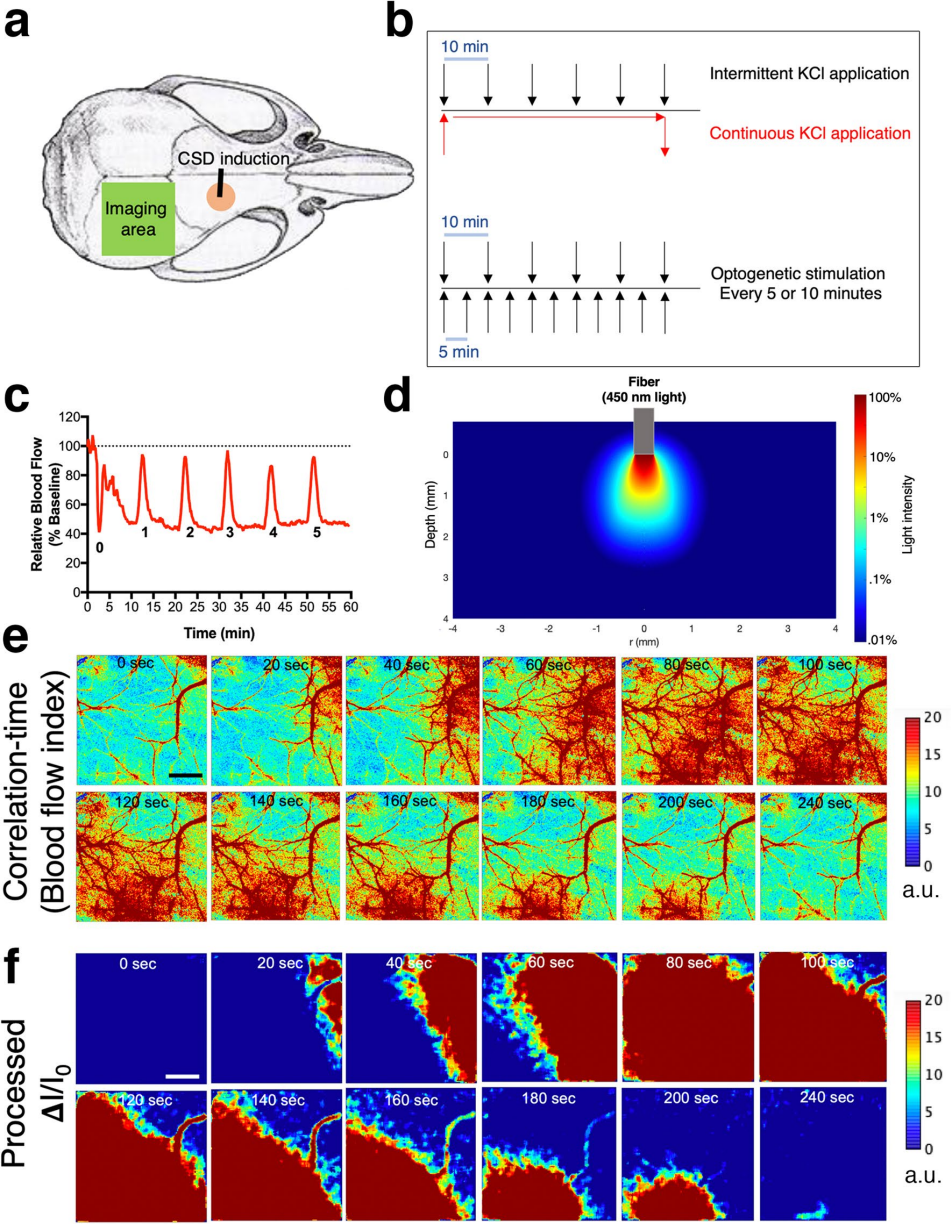
Experimental design. (**a**) Skull model showing the thinned skull preparation (imaging area) and the burr holes for KCl application or probe placement for optogenetic stimulation. (**b**) Timeline showing KCl application or optogenetic stimulation in all four experimental groups. (**c**) A representative 1-h relative cerebral blood flow (rCBF) trace from an animal with an optogenetically induced CSD, showing the first triphasic CSD wave (marked as 0) and the subsequent biphasic waves (marked as 1–5). Measurement is done in a 0.25 mm^2^ square ROI inside the imaging window. All pixel rCBF values inside this ROI are averaged and plotted over time. (**d**) Intensity profile for 450 nm light originating from the optogenetic stimulation fiber placed (400 µm, NA 0.48) placed over mouse skull, by Monte Carrlo simulation as a function of distance from the fiber. (**e**,**f**) Representative spatiotemporal blood flow maps showing CSD wave propagation through the imaging window as a biphasic hyperemic transient. (**e**) Correlation-time images of a single CSD wave, a biphasic wave of hyperemia as it enters and exits the imaging window. Correlation-time images are computed from speckle contrast images and the illustrated values are proportional with blood flow. The higher flow is indicated with warmer colors. (**f**) Further processed images to show DI/I_o_ values with respect to baseline and after spatiotemporal filtering, reveal boundaries of the CSD wave more clearly, making execution of automated optical flow codes possible. Scalebars: 1 mm.

We induced recurrent CSD waves propagating in the anteroposterior direction through the imaging field with KCl or repetitive optogenetic stimulation (Fig. 1a–d). During those experiments, monitored oxygen saturation was above 96% and heart rate was within the range of 400–500 beats per minute, corresponding to normal values in metabolically uncompromised rodents. We assessed CSD propagation by monitoring the associated blood flow changes because this allowed evaluation of the entire imaging field pixel by pixel unlike electrophysiological recordings (Fig. 1e,f). We did not prefer voltage-sensitive dyes, as they required opening of the skull, which may modify the CSD propagation^21, 22^. We omitted the first-ever CSD triggered in each experiment and studied the five subsequent CSD waves, starting with the second one in order because the phases of hemodynamic changes with respect to baseline differ between the first and repeat CSDs as reported previously^15^,the first wave being always triphasic, whereas the subsequent ones are biphasic (Fig. 1c). Mean propagation velocity of the CSD waves was 3.9 ± 0.7 mm/min and each CSD was observed for 140 ± 48 s until they exited the window.

**Figure 2:**
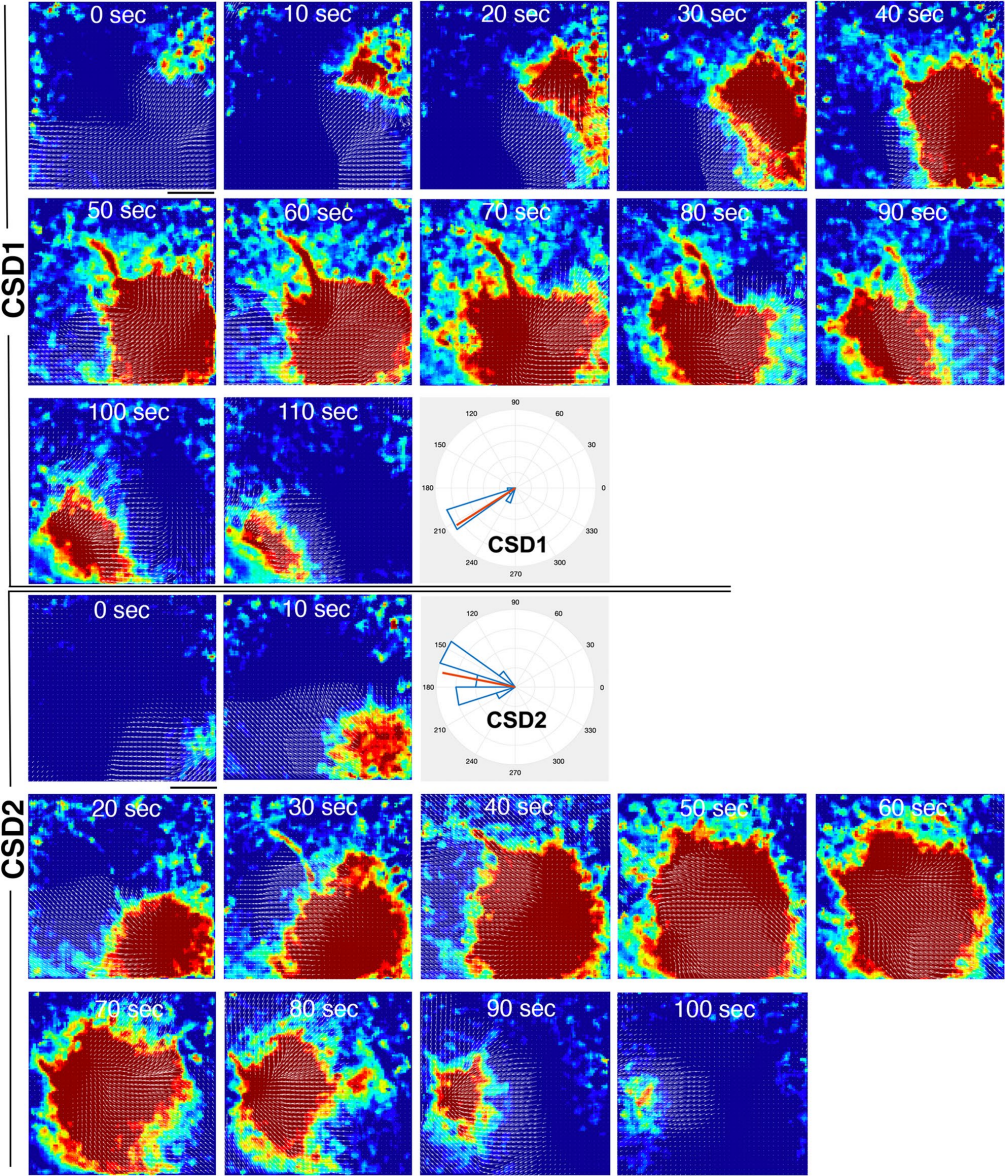
DI/I_o_ time series of two consecutive CSD waves from an animal with spontaneously triggered CSDs by continuous KCl application. Arrows indicate output of the optical flow analysis of pixel-wise angular velocity data. Distinctive propagation trajectories (SW vs. NW) of the two CSDs can be easily traced. Scalebars: 1 mm. Rose-plot histograms for CSD1 and CSD2 show the distribution of mean propagation vector angle throughout the time series of both experiments (Mean vector: 210 degrees for CSD1, 170 degrees for CSD2).

**Figure 3:**
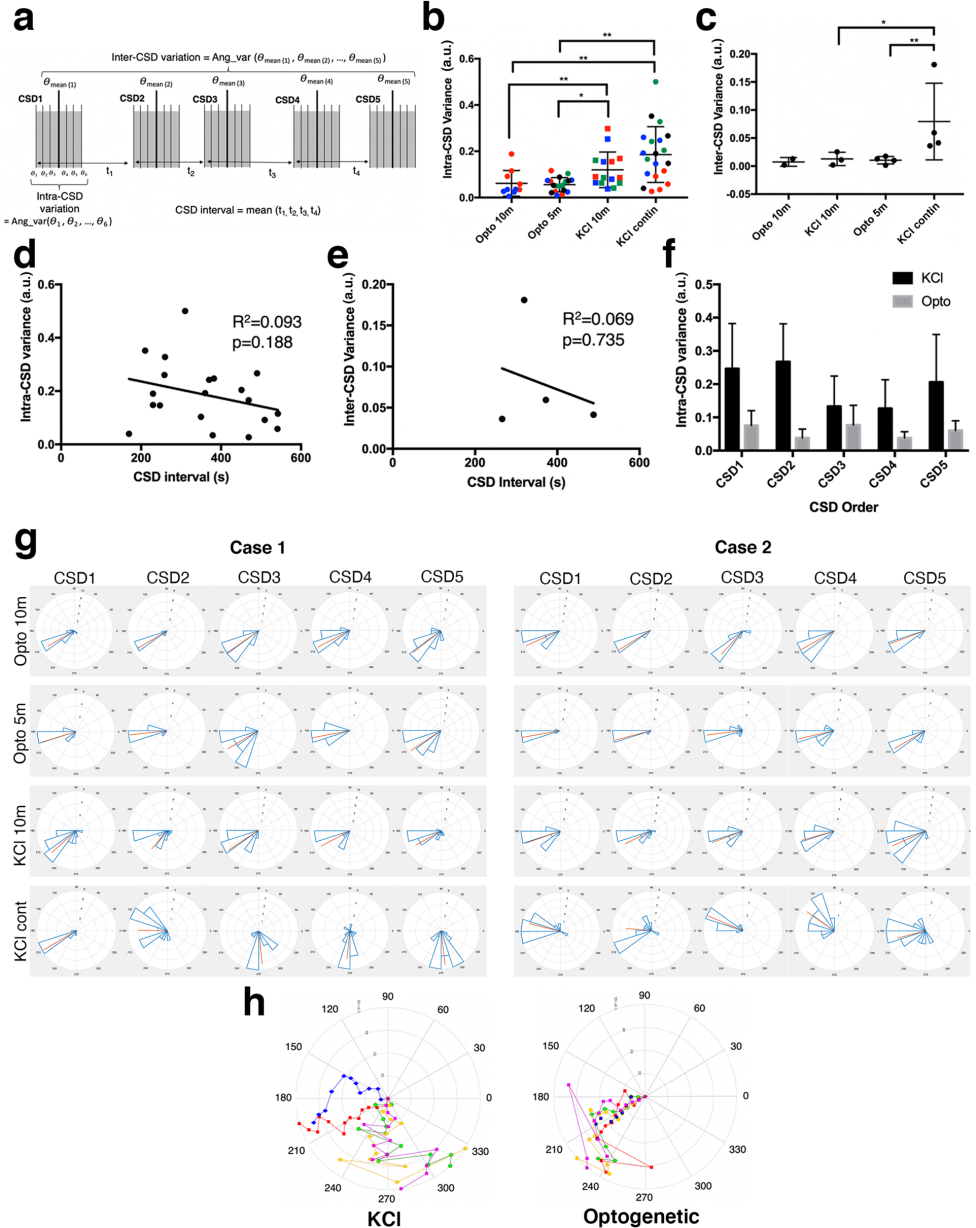
Comparison of the angular CSD variation across experimental groups and its association with the CSD interval and order. (**a**) Schematic showing the definitions of intra-CSD variation, inter-CSD variation and CSD intervals measured from the consecutively recorded 5 CSDs. Time between the start of consecutive CSDs is defined as t_1_, t_2_, t_3_ and t_4_. Angular average of all pixel-wise propagation vectors for each timepoint during the lifetime of a CSD is shown as θ_1_, θ_2_, … θ_n_ where n is the number of frame count of the time series. For illustration, each CSD is shown as if made of 6 frames. Mean of these 6 angular vectors (θ_mean_) is shown with a thick vertical bar. “Ang_var” is the function calculating the angular variation of the input vectors. (**b**) Intra-CSD variability is highest in case of CSDs triggered with continuous KCl application, followed by the group with intermittent (every 10 min) application of KCl. Each point represents the average angular variance of five CSDs from a single mouse. Each experiment is indicated with a different color. Optogenetically-triggered CSDs had less angular variation, regardless of the CSD interval. *p = 0.05, **p < 0.05. (**c**) Inter-CSD variation was higher in the continuous KCl application group, whereas the other groups showed no significant difference. Each point represents the mean of 5 CSDs from an animal. (**d**, **e**) Intra- and inter-CSD variances were compared to varying CSD intervals only from the continuous KCl application group because the CSD intervals were fixed in other groups. Each point represents the mean intra-CSD variance with the matching average CSD interval of 5 CSDs from an animal. Line shows the linear fit of the points, which was not significant and had low R^2^ (0.38 for inter-CSD, 0.22 for intra-CSD variance). (**f**) Intra-CSD variances of 5 consecutive CSDs in optogenetic stimulation and pooled KCl groups (i.e. regardless of the CSD interval), with respect to the CSD order. No significant association with the CSD order and intra-CSD variance was detected, suggesting that intra-CSD variance was similar across all consecutive CSDs independently of their order. The differences between KCl and optogenetic groups were significant for all CSD orders, except for the 3rd CSD (p = 0.22). (**g**) Rose plots showing distribution of propagation angles in all analyzed CSDs in two representative cases per experimental group. (**h**) The left rose plot shows the angular propagation trace of the 5 consecutive CSDs in a continuous-KCl applied animal, and the right rose plot shows the angular propagation trace in an animal with optogenetically (10-min intervals) triggered CSDs. Note the relatively less inter-CSD and intra-CSD propagation angle variability in the bottom plot.

Using automated optical flow tools, we calculated the pixel-wise propagation vectors of the CSD wave for each imaging frame in sequence (on average 14 frames recorded over ~ 2.3 min) and then constructed a 2D (x–y) propagation profile for every single CSD (Fig. 2). We could then quantify various parameters on the uniformity or variation of the propagation vectors within the same CSD wave (intra-CSD) or among successive CSD waves triggered in the same animal (inter-CSD) (Fig. 3a). We immediately noticed that in all experiments regardless of the technique, the mean propagation vector angle showed some degree of time-dependent fluctuation (range 17°–70°) such that the angle of vector changed as the wave progressed in the imaging field (Figs. 2, 3h). This quantitatively documents that the propagation of a single CSD is not perfectly concentric and symmetric with respect to its origin in the lissencephalic mouse brain. Omnibus tests showed that only 12% of total CSDs (n = 65) showed uniform temporal distribution of mean propagation vector directions as the wave progressed. This uniformity significantly varied with the CSD induction method: only 3% of the KCl-triggered CSDs (both continuous and intermittent KCl groups pooled) (n = 35) had uniform angular distribution in contrast to 23% of optogenetically triggered CSDs (n = 30) (χ^2^ = 4.524, p = 0.033) (Table 1).

**Table 1:** Various measurement parameters in experimental groups.

	KCl-continuous (n = 20)		KCl-10 min (n = 15)		Optogen-5 min (n = 20)		Optogen-10 min (n = 10)	
CSD interval (s)	361 ± 95		607 ± 1		300		600	
Mean propagation angle [95% confidence interval] (degrees)	− 157.84 [− 231.91 to − 83.77]		− 150.77 [− 161.07 to − 140.47]		− 166.16 [− 171.41 to − 160.91]		− 148.35 [2.02] [− 150.37 to − 146.33]	

	Intra-CSD	Inter-CSD	Intra-CSD	Inter-CSD	Intra-CSD	Inter-CSD	Intra-CSD	Inter-CSD
Standard deviation	0.69	0.37	0.46	0.14	0.32	0.13	0.32	0.11
Variance	0.25	0.07	0.12	0.01	0.06	0.01	0.06	0.01
Kurtosis	0.47	0.74	0.65	0.95	0.80	0.96	0.83	0.97
Percent with uniform distribution (Omnibus)	5		0		25		20	

We then wanted to systematically evaluate the propagation variability within the same CSD and among recurrent CSDs with regard to the CSD induction method. We quantified these heterogeneities as angular variations for each CSD (intra-CSD variation) and across different CSDs for each animal (inter-CSD variation). Both intra-CSD and inter-CSD variations were significantly higher when KCl-soaked cotton ball was left in place to trigger multiple CSD waves, compared to the other two methods (p < 0.05, Table 2) (Fig. 3b,c). The mean CSD interval with this continuous KCl application method was 361 ± 95 s. Even the two CSD waves immediately following each other had very different propagation directions, for instance, one wave propagating in north-northwest direction, the next one in south-southwest direction (Fig. 2). Intra-CSD and inter-CSD variations were significantly lower than were in the case of continuous KCl application (p < 0.05, Table 2), when epidural KCl was applied with 10-min intervals by removing the cotton ball after each CSD has been triggered (Fig. 3b,c). It should be noted that although we saw numerous CSD waves changing propagation direction within the imaging window in the continuous KCl application group, we did not observe re-entering or spiraling waves as in the gyrencephalic brains^4^, however, we cannot exclude the unlikely possibility that they might have done so after leaving the imaging window.

**Table 2:** Statistical comparisons between experimental groups.

	P-values: Kruskal Wallis ANOVA	P-values: Mann Whitney U test					
		Opto 10 m-KCl 10 m	Opto 10 m-Opto 5 m	Opto 10 m-KCl contin	KCl 10 m-Opto5m	KCl 10 m-KCl contin	Opto 5 m-KCl contin
Intra-CSD variance	< 0.01	0.02	> 0.90	< 0.01	< 0.01	0.11	< 0.01
Inter-CSD variance	0.02	> 0.90	> 0.90	0.05	0.13	> 0.90	0.03

On the other hand, optogenetically-triggered CSD waves with exactly 10-min or 5-min intervals had significantly lower intra and inter-CSD variations compared to the CSDs evoked by continuous or intermittent KCl application (p < 0.05, Table 2) without showing significant difference in angular propagation heterogeneity with respect to the CSD interval (Fig. 3b,c). We applied the optogenetic stimulus at 10-min intervals to match the conditions in KCl experiments, whereas the 5-min interval was used to see if the relative refractory period had an effect on propagation of the subsequent CSDs. Measures of kurtosis were used to evaluate the tailedness profile of propagation angle across groups (Table 1). The group with the highest angular variances, (recurrent CSDs triggered with continuous KCl application) had lower kurtosis, meaning greater extremity of deviations in tails of mean propagation angles, compared to the more homogeneous, less scattered angular propagation profile in optogenetically induced CSDs (Table 1, Fig. 3g,h).

Interestingly, although we detected a higher intra-CSD variance in the periodic 10-min KCl application group compared to optogenetically-triggered recurrent CSDs, inter-CSD variance was not higher in the 10-min KCl group (Fig. 3c). Inter-CSD variance only increased with continuous epidural KCl application. We did not find a significant correlation between CSD angular variances and CSD intervals (Inter-variance R^2^ = 0.069, p = 0.7354, Intra-variance: R^2^ = 0.094, p = 0.1885) (Fig. 3d,e). We also tested if the CSD order had any effect in angular variations, but we did not find any significant effect in this regard, in both KCl and optogenetic group of animals (Fig. 3f).

## Discussion

This study provides an unbiased and quantitative confirmation of the previous observations on heterogeneous and non-concentric propagation of CSD waves in both gyrencephalic and lissencephalic brains^4, 6–10, 12^, while also demonstrating how automated quantitative optical flow tools can be applied to this field. Our approach is methodologically advantageous because previous analyses were primarily based on manual annotation of CSD wave boundaries and time-dependent velocity change measurements, while automated optical flow analysis provides a faster and, importantly, unbiased alternative to manual strategies. Using these analysis tools, we show that the method of CSD induction (optogenetic vs. KCl) has an effect in the angular propagation variance of CSD waves in mice. Continuous epidural KCl application that triggers repetitive CSDs with varying intervals, caused the most heterogeneous, asymmetric, and non-concentric distribution of propagation vectors. When topical KCl was applied at 10-min intervals, we observed a higher intra-CSD variance, but not inter-CSD variance, compared to the optogenetically-triggered CSDs. Optogenetic CSDs had lower inter- and intra-CSD angular variation than KCl-induced CSDs independently of the CSD interval (5 vs 10 min). This suggests that CSD induction interval alone did not determine the spatiotemporal propagation direction. CSD intervals shorter than 5 min did not appear to particularly affect intra- as well as inter-CSD heterogeneity in the continuously KCl applied group despite the fact that the amplitudes of CSDs ignited after a shorter latency (i.e. < 300 s) were modified by the relative refractory period induced by the preceding CSD. Triggering CSDs optogenetically at intervals shorter than 5 min was avoided because it would not guarantee to induce a new full-blown CSD wave, limiting standardization of the CSD-to-CSD comparisons.

Heterogeneity in CSD propagation angle may depend on the cytoarchitecture of cortical zones being invaded by the wave front^10^, orientation of vascular structures^8^, differences in intrinsic excitability across the cortical tissue as well as variations in the location where CSD is triggered^7, 8, 23^. The small variability in CSD propagation with optogenetic stimulation compared to the KCl application suggests that heterogeneity arises at least partially at the induction site. In optogenetic stimulation, a fiber placed over the skull delivers blue light through a cannula forming an approximately 1 mm-diameter spot of effective illumination^24^ and triggers CSD uniformly across trials without substantial K^+^ rise at the time of ignition^22^, whereas in case of stimulation with KCl, a KCl-soaked cotton-ball is placed epidurally through a 1.5 mm-diameter burr hole to increase the intracortical extracellular concentration of K^+^ for depolarizing sufficient number of neurons. The minimum CSD ignition volume^25^^,^ which is estimated to be 0.005–0.012 mm^3^ can be anywhere inside this high [K^+^]_o_ zone under the cotton ball. A physiological mechanism determining this focal point could be the astrocyte-mediated regulation of local extracellular potassium in the cortex^26^ by transporting K^+^ ions from high to lower concentration areas; in our case, first from the subarachnoid space to the brain interstitium and then to microcirculation. In case of prolonged KCl application, sustained depolarization of astrocytes may reduce this K^+^ siphoning function and may shift the K^+^ trigger point to the peripheral zones of the burr hole where astrocytes are yet not so depolarized. An extreme example of this phenomenon is the local neuroglial necrosis seen after extended application of high molar KCl^27, 28^. Indeed, optogenetic stimulation, in addition to being consistently limited to a fixed tissue underneath the optrode, also ignites CSD at a time when extracellular K^+^ levels are not so high to depolarize nearby astrocytes (in contrast to KCl application), hence, astrocytes can suck up released K^+^ from depolarized neurons and uniformly dissipate it^22^. Consequently, both CSD ignitions at the same point with repeated stimulations and homogenous dissipation of released K^+^ necessary for the propagation of CSD may account for the small angular variation seen with optogenetic stimulation. In addition to shifting the ignition point with KCl, slowly recovering cortical areas from the refractory period may also divert the propagation angle toward the neighboring faster recovering areas and, hence, contribute to heterogeneity. When KCl-soaked cotton balls are applied with 10-min intervals, glia limitans astrocytes may better recover from depolarization and cortical neurons from the relative refractory period before the subsequent application, partly reducing the propagation heterogeneity. The other factors discussed above may also contribute to the residual heterogeneity in this group.

Optogenetic triggering of CSDs, being non-invasive, has higher translational value compared to conventional methods, and is especially suitable for longitudinal studies requiring repeated stimulations. Brief exposure of the cortex to light in ChR2-expressing mice induces CSDs without causing injury and significant metabolic stress to the brain tissue at the stimulation point unlike KCl application. Making them even more physiologically relevant, optogenetic stimulus initially depolarizes an aggregate of neurons before astrocytes are depolarized by released K^+^, similar to the hypothesized mechanisms for spontaneous CSD generation in healthy brain^1, 22, 29^. The more homogenous propagation of optogenetically-triggered CSDs compared to KCl-evoked ones offers another advantage of the optogenetic CSD models by minimizing variation in tissue outcomes caused by inconsistent exposure to recurrent CSDs.

It should be noted that our analysis is based on the hemodynamic changes associated with CSDs and assumes that the electrophysiological changes propagate the same way. However, the electrophysiological-hemodynamic coupling may be additionally disturbed during recurrent CSDs and contribute to the propagation angle heterogeneity. This possibility warrants future studies using high-density multielectrode arrays or voltage-sensitive dye imaging.

In conclusion, this study establishes the heterogeneity of CSD angular propagation even in lissencephalic brains, which can promote interest in the propagation of CSDs in the gyrencephalic human brain, which are commonly seen in migraine, stroke, traumatic brain injury and subarachnoid hemorrhage^6, 30, 31^. Additionally, this suggests caution for CSD research in rodents, especially if repetitive CSDs are to be studied, that the propagation heterogeneities may result in local variations in experimental findings.

## Data Availability

The data sets generated during and/or analyzed during the current study, raw image files, custom MATLAB codes used to process and analyze data are available from the corresponding author upon request.

## References

[1] Pietrobon D, Moskowitz MA (2014). Chaos and commotion in the wake of cortical spreading depression and spreading depolarizations. Nat. Rev. Neurosci..

[2] Martins-Ferreira H, Nedergaard M, Nicholson C (2000). Perspectives on spreading depression. Brain Res. Brain Res. Rev..

[3] Martins-Ferreira H, de Castro GO (1966). Light-scattering changes accompanying spreading depression in isolated retina. J. Neurophysiol..

[4] Santos E (2014). Radial, spiral and reverberating waves of spreading depolarization occur in the gyrencephalic brain. Neuroimage.

[5] Dreier JP (2017). Recording, analysis, and interpretation of spreading depolarizations in neurointensive care: Review and recommendations of the COSBID research group. J. Cereb. Blood Flow Metab..

[6] Woitzik J (2013). Propagation of cortical spreading depolarization in the human cortex after malignant stroke. Neurology.

[7] Kumagai T (2011). Distinct spatiotemporal patterns of spreading depolarizations during early infarct evolution: Evidence from real-time imaging. J. Cereb. Blood Flow Metab..

[8] Kaufmann D (2017). Heterogeneous incidence and propagation of spreading depolarizations. J. Cereb. Blood Flow Metab..

[9] Chen S (2006). Time-varying spreading depression waves in rat cortex revealed by optical intrinsic signal imaging. Neurosci. Lett..

[10] Eiselt M (2004). Inhomogeneous propagation of cortical spreading depression-detection by electro- and magnetoencephalography in rats. Brain Res..

[11] Li B, Chen S, Yu D, Li P (2015). Variation of repetitive cortical spreading depression waves is related with relative refractory period: A computational study. Quant. Biol..

[12] Li B, Chen S, Li P, Luo Q, Gong H (2014). Refractory period modulates the spatiotemporal evolution of cortical spreading depression: A computational study. PLoS ONE.

[13] Milakara D (2017). Simulation of spreading depolarization trajectories in cerebral cortex: Correlation of velocity and susceptibility in patients with aneurysmal subarachnoid hemorrhage. NeuroImage Clin..

[14] Takizawa T (2019). Non-invasively triggered spreading depolarizations induce a rapid pro-inflammatory response in cerebral cortex. J. Cereb. Blood Flow Metab..

[15] Ayata C (2004). Pronounced hypoperfusion during spreading depression in mouse cortex. J. Cereb. Blood Flow Metab..

[16] Boas DA, Dunn AK (2010). Laser speckle contrast imaging in biomedical optics. J. Biomed. Opt..

[17] Afrashteh N, Inayat S, Mohsenvand M, Mohajerani MH (2017). Optical-flow analysis toolbox for characterization of spatiotemporal dynamics in mesoscale optical imaging of brain activity. Neuroimage.

[18] Stujenske JM, Spellman T, Gordon JA (2015). Modeling the spatiotemporal dynamics of light and heat propagation for invivo optogenetics. Cell Rep..

[19] Haleh S, Hirac G, Frédéric P (2017). Optical properties of mice skull bone in the 455- to 705-nm range. J. Biomed. Opt..

[20] Berens P (2009). CircStat : A MATLAB toolbox for circular statistics. J. Stat. Softw..

[21] Ayata C (2013). Pearls and pitfalls in experimental models of spreading depression. Cephalalgia.

[22] Chung DY (2019). Determinants of optogenetic cortical spreading depolarizations. Cereb. Cortex.

[23] Kroos JM, Diez I, Cortes JM, Stramaglia S, Gerardo-Giorda L (2016). Geometry shapes propagation: Assessing the presence and absence of cortical symmetries through a computational model of cortical spreading depression. Front. Comput. Neurosci..

[24] Dhakal KR (2014). Non-scanning fiber-optic near-infrared beam led to two-photon optogenetic stimulation in-vivo. PLoS ONE.

[25] Tang YT (2014). Minimum conditions for the induction of cortical spreading depression in brain slices. J. Neurophysiol..

[26] Bellot-Saez A, Kékesi O, Morley JW, Buskila Y (2017). Astrocytic modulation of neuronal excitability through K+ spatial buffering. Neurosci. Biobehav. Rev..

[27] Nedergaard M, Hansen AJ (1988). Spreading depression is not associated with neuronal injury in the normal brain. Brain Res..

[28] Plumier JCL, David JC, Robertson HA, Currie RW (1997). Cortical application of potassium chloride induces the low-molecular weight heat shock protein (Hsp27) in astrocytes. J. Cereb. Blood Flow Metab..

[29] Kager H, Wadman WJ, Somjen GG (2002). Conditions for the triggering of spreading depression studied with computer simulations. J. Neurophysiol..

[30] Ayata C, Lauritzen M (2015). Spreading depression, spreading depolarizations, and the cerebral vasculature. Physiol. Rev..

[31] Bouley J, Chung DY, Ayata C, Brown RH, Henninger N (2018). Cortical spreading depression denotes concussion injury. J. Neurotrauma.

